# Neoadjuvant Immunotherapy in Head and Neck Cancers: A Paradigm Shift in Treatment Approach

**DOI:** 10.3390/biomedicines12102337

**Published:** 2024-10-14

**Authors:** Alessia Zotta, Maria Luisa Marciano, Francesco Sabbatino, Alessandro Ottaiano, Marco Cascella, Monica Pontone, Massimo Montano, Ester Calogero, Francesco Longo, Morena Fasano, Teresa Troiani, Fortunato Ciardiello, Fabiana Raffaella Rampetta, Giovanni Salzano, Giovanni Dell’Aversana Orabona, Luigi Califano, Franco Ionna, Francesco Perri

**Affiliations:** 1 Medical Oncology, Department of Precision Medicine, Università degli Studi della Campania Luigi Vanvitelli, 80128 Naples, Italy; alessia.zotta@studenti.unicampania.it (A.Z.); morena.fasano@unicampania.it (M.F.); teresa.troiani@unicampania.it (T.T.); fortunato.ciardiello@unicampania.it (F.C.); 2 Head and Neck Oncology Unit, Istituto Nazionale Tumori di Napoli IRCCS “G. Pascale”, 80131 Naples, Italy; m.pontone@istitutotumori.na.it (M.P.); m.montano@istitutotumori.na.it (M.M.); e.calogero@istitutotumori.na.it (E.C.); rampetta.fabiana@libero.it (F.R.R.); f.perri@istitutotumori.na.it (F.P.); 3 Medical Oncology Department, Università degli Studi di Salerno, 84084 Salerno, Italy; fsabbatino@unisa.it; 4 Abdominal Oncology Unit, Istituto Nazionale Tumori di Napoli IRCCS “G. Pascale”, 80131 Naples, Italy; a.ottaiano@istitutotumori.na.it; 5 Anesthesiology and Pain Therapy Unit, Università degli Studi di Salerno, 84084 Salerno, Italy; mcascella@unisa.it; 6 Maxillofacial and ENT Surgery Unit, Istituto Nazionale Tumori di Napoli IRCCS “G. Pascale”, 80131 Naples, Italy; f.longo@istitutotumori.na.it (F.L.); f.ionna@istitutotumori.na.it (F.I.); 7 Head and Neck Section, Department of Neurosciences, Reproductive and Odontostomatological Science, Federico II University of Naples, 80138 Naples, Italy; giovannisalzanomd@gmail.com (G.S.); giovanni.dellaversanaorabona@unina.it (G.D.O.); luigi.califano@unina.it (L.C.)

**Keywords:** immunotherapy, neoadjuvant, checkpoint inhibitors, head and neck squamous cell carcinoma, tumor mutational burden

## Abstract

Checkpoint inhibitors (ICIs) have demonstrated substantial efficacy in the treatment of numerous solid tumors, including head and neck cancer. Their inclusion in the therapeutic paradigm in metastatic lines of treatment has certainly improved the outcomes of these patients. Starting from this assumption, numerous studies have been conducted on ICIs in other earlier disease settings, including studies conducted in patients in neoadjuvant settings. However, how many and which studies are truly significant? Can they lay concrete foundations for further future studies and therefore allow us to continue to have this interesting future perspective? Through a review of the existing literature, coupled with insights gleaned from clinical practice and from the main recently published studies, we aim to examine the therapeutic potential of ICIs in patients affected by head and neck cancer in a neoadjuvant treatment setting and encourage researchers to set up successful future clinical trials.

## 1. Background

*Immunotherapy and ICIs* Immunotherapy has emerged as a revolutionary paradigm in many cancer treatments by exploiting the intricate interplay between the immune system and cancer cells. Unlike conventional therapies that directly target tumor cells, immunotherapy aims to booster the body’s immune response, enabling it to recognize and to destroy cancer cells more effectively.

Head and neck squamous cell carcinoma (HNSCC) accounts for 4–6% of all cancers, with 6000 newly diagnosed patients every year in the United States. HNSCC is the seventh most common cancer worldwide with an annual incidence of approximately 700,000 and a mortality rate estimated at 350,000 in 2018 [1]. This tumor occurs in various sites, such as the oral cavity, larynx, and pharynx, due to the action of two main risk factors, smoking and alcohol consumption. Nevertheless, in the last 20 years, we have observed an increasing number of newly diagnosed human papilloma virus (HPV) infection-associated cancers [2]; their incidence has been increasing recently, and HPV 16 prevalence is >80% among HPV-infected Oropharyngeal squamous cell carcinoma (OPSCC) patients [3].

HSNCCs are easily accessible to clinical examination since they are visible by fibroscopy: this allows us to monitor them in real time without using daily radiological investigations, and to perform biopsies of both primary tumor and metastatic lymph nodes [4]. The treatment of HNSCC is characterized by an integration of different types of therapies, such as chemotherapy, radiotherapy, traditional surgery, targeted therapy, and, more recently, immunotherapy with administration of checkpoint inhibitors including anti-Programmed death receptor 1 (PD-1) pembrolizumab and nivolumab. The latter was approved in 2016 for recurrent/metastatic (R/M) HSNCC that progresses after chemotherapy failure. In addition, many other trials have investigated or are currently investigating checkpoint inhibitors targeting anti-PD-1 or its ligand programmed death receptor ligand 1 (PD-L1) as well as cytotoxic T lymphocyte associated protein 4 (CTLA-4) in both metastatic and early-stage settings [5].

The Cancer Genome Atlas’ (TCGA) data show that HNSCC is one of the most immune-active tissue after lung adenocarcinoma and renal cell carcinoma: it is commonly infiltrated by immune cells with a strong immunosuppressive phenotype, like M2 macrophages, T regulatory cells, and myeloid-derived suppressor cells (MDSCs) [6,7]. Immune checkpoint inhibitors represent a potential key to overcome an immunosuppressive tumor phenotype, unleashing the immune system’s ability to target and destroy cancer cells. As a result, targeting the immune system and tumor microenvironment may be a new therapeutic strategy for many cancers [8]. Immune checkpoints are a part of the protein ligand receptor that controls T cells activation; so, the application of ICIs to block the role of immune checkpoints can promote the release of T cells, increase the anti-tumor response, and enhance tumor cell clearance and immune monitoring. PD-1 is a membrane-spanning protein part of the CD28 group of T cell costimulatory receptors, found in numerous immune cells, predominantly cytotoxic T cells [9,10]. It interacts with its ligands (PD-L1 and PDL2), leading to anergy of T cell and immune-escape [11].

Deep understanding of the mechanisms and cell types governing immunotherapy response in head and neck squamous cell carcinoma (HNSCC) is still in its infancy. A recent analysis of neoadjuvant PD-1 or combined PD-1/CTLA-4 blockade indicated an early expansion of intra-tumoral CD8^+^ tissue-resident memory cells and blood-derived clonotype expansion, particularly following combination therapy. However, the dynamics of tumor microenvironment cellular composition between responder and non-responder patients were not investigated [12].

Pembrolizumab has been the first PD-1 inhibitor to be approved for R/M HNSCC. It showed a response rate of 18% (KEYNOTE-012 Trial) [13], allowing for its further implementation in further studies, such as the KEYNOTE 048 Trial. In this trial, the effectiveness of pembrolizumab alone or pembrolizumab combined with chemotherapy was compared to that of traditional chemotherapy in platinum refractory patients, resulting in an improved overall survival in the group that received pembrolizumab with or without chemotherapy as compared to traditional chemotherapy (13 months vs. 10.7 months, HR 0.77, *p* = 0.0034). Thus, this has become a new standard of care in the first-line treatment of R/M HNSCC [14].

On the other hand, the CHECKMATE 141 trial has been the first phase III trial which demonstrated the superiority of the PD-1 inhibitor nivolumab as compared to standard chemotherapy for R/M HNSCC in the second line of treatment after a progression disease in less than 6 months from a chemotherapy regimen. The results showed that nivolumab was significantly better than standard chemotherapy, with an increased median survival time of 2.4 months and a 20% higher survival rate [15].

In HSNCC, this is a rapidly evolving theme, both as immunotherapy alone or in combination with other drugs or techniques. Thus, immunotherapy stands as a promising frontier in the treatment of HNSCC, offering a tailored approach that harnesses the body’s own immune system to fight against cancer. While traditional therapies have shown limitations in efficacy and tolerability, immunotherapy represents a paradigm shift in cancer treatment, offering durable responses and improved outcomes for patients. With ongoing research and advancements in understanding tumor immunology, the future holds great potential for further optimizing immunotherapeutic strategies, ultimately reshaping the landscape of HNSCC management and providing new hopes for patients worldwide.

## 2. Neoadjuvant Immunotherapy in Locally Advanced (LA) HNSCC

### 2.1. Rationale

In the realm of oncology, the treatment landscape for head and neck cancer has witnessed remarkable advancements in recent years. Among these breakthroughs, neoadjuvant immunotherapy has emerged as a promising approach, revolutionizing the conventional paradigms of cancer treatment. By harnessing the power of the body’s immune system, neoadjuvant immunotherapy holds the potential to enhance therapeutic outcomes, particularly in the context of head and neck malignancies.

Recently, new clinical trials have been focusing on non-metastatic disease, with particular attention towards LA HNSCC, and several groups have explored the impact of delivering ICIs in the neoadjuvant setting [16,17,18,19,20,21]. Most of these trials have focused on non-HPV (Human Papilloma Virus)-related disease. The latter shows the worst outcomes, but it is expected to benefit from treatment intensification. Neoadjuvant therapy offers many advantages since it applies to naive patients, whose body has never been impacted by surgery, radiation therapy or chemotherapy. As a result, the patients’ immune system can be primed with the tumor and lymph nodes that are still in place to mount an efficient immune response [22].

LA HNSCC is defined as either stage III or IV oral cavity, larynx, hypopharynx and p16-negative oropharyngeal cancer, o T3–4/N0–3 and T0–4/N1–3 p16-positive oropharyngeal cancer according to the TNM 8th edition of American Joint Committee on Cancer (AJCC); it often requires postoperative chemo-radiation with a high grade of toxicities and/or induction chemotherapy. Disease-free survival (DFS) after 2 years is about 70% with standard therapies. Potential options for locally advanced HNSCC are either surgery followed by adjuvant chemo-radiotherapy C(RT) or primary chemo-radiotherapy CRT alone, with a surgical option that includes reconstruction plus risk-adapted postoperative RT or CRT. CRT is the standard of treatment in non-resectable patients, and it is indicated in resectable patients when the anticipated functional outcome and/or the prognosis is so poor that mutilating surgery is not justified [5]. Despite these intensive treatments, recurrence of disease is one of the most important key causes of treatment failure. One of the first neoadjuvant approaches (defined as a systemic therapy prior to surgery) has been induction using a chemotherapeutic regimen which includes docetaxel, 5FU and cisplatin (TPF), firstly explored in oral cavity cancers. An improvement in overall survival (OS) was not achieved. However, there was a positive impact on surgical performance as compared to an upfront surgical approach [23,24], even in the cases of cervical node involvement [24]. Nowadays, the surgical approach remains the treatment of choice to be primarily performed in patients with surgically resectable disease; alternatively, if surgery cannot be performed upfront or there are high-risk factors for relapse, a CRT approach would be desirable [25,26].

Recently, the concept of neoadjuvant treatment has been revisited in several cancer types based on the implementation of immunotherapeutic drugs into upfront settings, with the aim of activating immunologic effector mechanism to kill cancer cells. In the context of HNSCC, neoadjuvant immunotherapy has emerged as a promising strategy to increase the effectiveness of conventional treatments and improve patient outcomes. By initiating immunotherapy prior to surgery or radiation, clinicians seek to prime the immune system, enhance tumor recognition, and potentially downsize tumors, making them more amenable to subsequent treatment modalities. Although previous research did not establish a survival advantage for neoadjuvant chemotherapy in patients with locally advanced HNSCC, certain studies reported pCR rates between 10% and 27%, indicating an improved overall survival for those with pCR [12,23,24,25,26,27,28,29,30,31,32]. Moreover, earlier neoadjuvant studies involving immune checkpoint inhibitors in locally advanced HNSCC demonstrated low pCR rates varying up to 6.7% [16], implying that combinatorial neoadjuvant therapies are needed to achieve deeper tumor regression during the neoadjuvant treatment.

Data from pre-clinical models suggest that priming an immune response may also be superior in the neoadjuvant setting [27], which leads to speculation about early targeting of immunity mechanisms in treatment-naïve patients. 

In head and neck cancer, the tumor microenvironment is characterized by immunosuppressive mechanisms, such as upregulation of immune checkpoint molecules like PD-L1 and CTLA-4. Utilizing immunotherapy following biopsy confirmation of disease, but preceding definitive surgery, provides a transitional phase of treatment to administer therapy and evaluate the clinical, radiological, and biological responses. Sequential samplings allow researchers to correlative investigations aimed at comprehending shifts in tumor-immune parameters and enable a correlation with the response. Multi-dimensional flow cytometry, immunohistochemistry, and multiplexed immunofluorescence can be conducted to measure immune cell populations and patterns of immune checkpoint receptor expression, with the latter offering insights into spatial tumor–immune cell interactions. Whole-genome and RNA sequencing platforms can be employed to elucidate genomic factors influencing immune cell activity, aiding in neo-antigen prediction modeling and protein expression analysis. Moreover, T cell receptor (TCR) clonotyping can identify distinct gene rearrangement sequences arising in response to antigen presentation in infiltrating lymphocytes within a specific tumor, while extra- or intracellular cytokine levels can be assessed to comprehend immune cell signaling. These methodologies can be collectively interpreted to grasp the dynamic and intricate tumor immune network and its modulation in reaction to immunotherapy administration.

ICIs are effective both in platinum-refractory and in platinum-sensitive/naive advanced HNSCC patients. Investigating new neoadjuvant approaches with immune-based therapies appears to be a promising approach since:

(1) It would lead to an early selection of treatment responders leading to an optimization of the surgical approach, also in terms of positive resection margin.
(2) It would facilitate the de-escalation of adjuvant post-operative radiation and/or monotherapy in surgical patients too.
(3) It would potentially downstage tumors in previously non-resectable disease, allowing the disease to be respected, and, at the same time, providing early systemic therapy to address the risk of distant metastatic spread.
(4) Lastly, it can synergize with conventional treatment modalities, such as chemotherapy and radiation therapy, to achieve enhanced antitumor effects. Preclinical and clinical studies have demonstrated that combining immunotherapy with standard treatments can overcome treatment resistance, improve response rates, and prolong survival in patients with head and neck cancer. This multimodal approach harnesses the strengths of different treatment modalities while minimizing their individual limitations.

Despite all these potential advantages, the constraints of an innovative, preoperative approach utilizing immunotherapy must be considered; administering preoperative therapy of any sort before definitive surgical removal is not without some hazard, as immune-mediated adverse effects can be severe with the worry of postponing curative surgery. Of particular interest has been the concept of accelerated progression (or hyper-progression), where patients experience hastened tumor growth kinetics following anti-PD-1/PD-L1 exposure [28]. The influence of alterations in the tumor immune microenvironment on hemostatic effects after immunotherapy exposure is uncertain. Additionally, immunomodulation could have implications regarding post-operative wound healing. While these latter concerns remain, largely speculative and initial studies indicate that preoperative immunotherapy is safe [29].

Several clinical trials have explored the efficacy and safety of neoadjuvant immunotherapy in head and neck cancer. Notably, studies investigating immune checkpoint inhibitors such as pembrolizumab and nivolumab have demonstrated promising results in terms of tumor response rates, PFS, and OS. Additionally, neoadjuvant immunotherapy has shown potential to induce immune activation within the tumor microenvironment, paving the way for more durable treatment responses and improved long-term outcomes.

### 2.2. Clinical Trials

The diversity of immune cells plays a crucial role in influencing how effectively cancer progresses and responds to treatment.

In a phase II trial (NCT02296684), it was observed that 45% of patients receiving two doses of neoadjuvant pembrolizumab exhibited substantial pathological tumor response (pTRs) (≥50%). Analysis of single cells from 14 tumor biopsies, including six matched pre- and post- neoadjuvant treatment samples, revealed that responsive tumors harbored expanded populations of exhausted CD8^+^ tumor-infiltrating lymphocytes (TILs) with a tissue-resident memory phenotype. These cells displayed high cytotoxic potential (CTX^+^) and expressed ZNF683, a marker associated with tissue-resident memory. pTRs after five weeks of PD-1 blockade were characterized by the activation of pre-existing CTX^+^ZNF683^+^CD8^+^ TILs, leading to a reduction in viable tumor tissue and associated tumor antigens. Successful responses were linked to elevated numbers of CD103^+^PD-1^+^CD8^+^ T cells infiltrating lesions before treatment. Meanwhile, the rejuvenation of non-exhausted persistent clones and clonal replacement were observed to a lesser extent. In contrast, baseline TMEs of non-responders showed a relative scarcity of ZNF683^+^CTX^+^ TILs and subsequent accumulation of highly exhausted clones. In HNSCC cases, the reinvigoration of pre-existing ZNF683^+^CTX^+^ TILs stand out as a primary mechanism of response following neoadjuvant treatment [12,30].

Another similar trial by Sequi Ren et al. underscored the significance of intratumoral CD103^+^CD8^+^ TILs density in determining the efficacy of neoadjuvant chemo-immunotherapy (NACI). This investigation revealed that individuals with a heightened concentration of intratumorally CD103^+^CD8^+^ TILs displayed a heightened efficacy in response to NACI for advanced HNSCC. Additionally, these cells showcased a robust cytotoxic behavior against tumor cells both in vitro and in vivo. Furthermore, the integration of CPS with the levels of CD103^+^CD8^+^ TILs in a composite predictive framework improved diagnostic accuracy, carrying potential implications for tailored patient consultation and the selection of chemo-immunotherapy treatments. Consequently, by delving into the intricate molecular processes governing the formation, functionality, and therapeutic advantages of intratumoral CD103^+^CD8^+^ TILs, we envisaged strategies that could steer the direction of personalized precision medicine in NACI [31,32].

In fact, the authors revealed that 40.0% (8/20) of patients exhibiting a high density of CD103^+^CD8^+^ TILs were at stages T1–T2, whereas only 21.1% (4/19) of those with a low density were in the same stages, indicating a greater infiltration of CD103^+^CD8^+^ T cells in T1–T2 primary tumors. Similarly, the authors observed that 21.1% (4/19) of patients with low CD103^+^CD8^+^ TILs density and 35.0% (7/20) of those with high density were classified as clinical stage III. Notably, 12 (70.6%) patients without lymph node metastasis were identified as having a high density of CD103^+^CD8^+^ TILs, whereas only 26.3% (5/19) of those with low density had no lymph node metastasis. Among patients in the poorly differentiated stage, merely 12.5% (1/8) had a high density of CD103^+^CD8^+^ TILs. Conversely, 81.3% (13/16) of patients with good differentiation exhibited high density of CD103^+^CD8^+^ TILs.

From April to December 2020, Zhang et al. [33] treated 20 patients affected by locally advanced HSNCC with the combination of camrelizumab and apatinib (an anti-VEGFR) before surgery. The safety of neoadjuvant camrelizumab and apatinib was evaluated as a primary outcome. Analysis of the results demonstrated that preoperative combination of camrelizumab and apatinib without chemotherapy can be safe and effective in patients with OSCC by achieving a major pathological response (MPR) in 40% of patients and confirming the safety profile obtained in previous reports in advanced cancer cases [24,34,35].

Another recent study reported a high rate of biopsy-proven pathological complete response (pCR) (52%) of the patients with LA HNSCC following administration of the anti-PD-L1 durvalumab plus anti-CTLA-4 tremelimumab and platinum-based chemotherapy [36].

Moreover, the obtained toxic profile from camrelizumab and apatinib appears to be more manageable and safer as compared to other preoperative chemotherapy regimens employed in OSCC trials, such as those utilizing TPF regimen or targeted therapies [37,38,39]. The latter often resulted in high rates of severe adverse events. Although a higher risk of bleeding associated with anti-VEGF(R) therapies is expected from small molecules such as apatinib, larger trials are warranted to conclusively determine the impact of preoperative anti-PD-1 plus anti-VEGFR therapy on surgical outcomes.

Incorporating stereotactic body radiation (SBRT) therapy alongside neoadjuvant immunotherapy is another approach which is currently investigated, especially in the recurrent/metastatic setting, but also in the locally advanced one.

In a phase Ib trial involving immuno-radiotherapy administered prior to surgery, 21 patients with newly diagnosed locally advanced HPV-positive and HPV-negative HNSCC received neoadjuvant SBRT. This was administered at either a cumulative dose of 40 Gy over five sessions or 24 Gy across three sessions within a week, with or without the addition of nivolumab, before undergoing definitive surgical removal of the tumor. As result, an impressive 86% rate of major pathological response (MPR) was achieved [40]. The authors concluded that that radiation delivered only to the gross tumor volume combined with immunotherapy was safe, resulting in a high rate of mPR [21].

In a recent phase II trial conducted by Kim et al., 31 patients with diagnosed R/M unresectable HNSCC were administered intravenous durvalumab in combination with tremelimumab every four weeks for four cycles, followed by durvalumab alone every four weeks. After completing one cycle of the durvalumab/tremelimumab regimen, proton therapy was delivered at a total dose of 25 Gy in daily fractions of 5 Gy to one of the measurable lesions. With a follow-up period of 8.6 months, the outcomes showed that the overall response rate (ORR) was 22.6%, including, interestingly, one complete response upon all target lesions (also lesions which were out of the irradiation fields. The primary tumor and/or lymph nodes were not always irradiated, as distant metastases were also irradiated at choice of the experimenter. Moreover, among the 23 evaluable patients who completed proton therapy, the ORR was 30.4%, with a median OS of 11.1 months. The authors concluded that neoadjuvant immunotherapy (with ICI) added to proton therapy was not only able to reach interesting and good activity and efficacy, but it was also capable of eliciting an abscopal effect, probably driven by the combination off ICI and proton therapy [41].

Liu et al. carried up a retrospective analysis upon 273 patients affected by metastatic de novo nasopharyngeal carcinoma treated with neoadjuvant chemo-immunotherapy followed by radiation therapy or alternative immune-chemotherapy alone. All patients received cisplatin-based chemotherapy plus camrelizumab. After chemo-immunotherapy, 178 patients received intensity-modulated radiotherapy (IMRT) to the nasopharynx and neck, while the remaining 95 patients stopped chemotherapy and only the responders continued with camrelizumab. As a result, disease progression occurred in just 25.6% of patients who received radiotherapy, compared to 55.7% of patients who did not have radiotherapy (*p* < 0.001). Moreover, with a median follow-up time of 18 months, patients with immune-chemotherapy plus subsequent radiotherapy had higher 1-year progression-free survival (80.6% vs. 65.1%, *p* < 0.001) and overall survival (98.3% vs. 89.5%, *p* = 0.001) than those treated with immune-chemotherapy alone [40]. 

In a case report recently published by Chen et al., a 64-year-old man affected by SNSCC (squamous sino-nasal cancer) treated by multi-protocol exploration with neoadjuvant tislelizumab plus chemotherapy with the following regimen, docetaxel 120 mg + cisplatin 120 mg (TP regimen) combined with tislelizumab immunotherapy 200 mg for two cycles, reached a complete remission. One year follow-up CT was performed and the results showed no tumoral lesions, and the comprehensive assessment of the tumor condition control was stable with no recurrence. Currently, clinical follow-ups are still performed and, to date, the patient has good compliance and tolerance, and no significant adverse reactions have occurred [42].

In a prospective, single-arm, open-label trial (Illuminate Trial), 20 patients affected by locally advanced oral squamous cell carcinoma (LAOSCC) received two cycles of NAICT (21 day each cycle), including intravenous albumin paclitaxel (260 mg/m^2^), cisplatin (75 mg/m^2^), and toripalimab (240 mg) in sequence on day 1 of each cycle, followed by radical surgery and risk-adapted adjuvant (chemo)radiotherapy. All of the enrolled patients reached a R0 resection. The MPR (major pathological response) rate was 60%, including a 30% pathological complete response. MPR was achieved in all four patients with a combined positive score of PD-L1 > 10, thus supporting future clinical trials with neoadjuvant immunotherapy in locally advanced oral squamous cell carcinoma [43].

In a phase II trial, 27 patients with histopathologically confirmed, resectable locally advanced laryngeal/hypopharyngeal squamous cell carcinoma and ECOG Performance Status 0–1 practiced three cycles of induction chemotherapy (paclitaxel 175 mg/m^2^ d1, cisplatin 25 mg/m^2^ d1–3) combined with PD-1 inhibitor (toripalimab 240 mg d1), thus resulting in encouraging activity, promising larynx preservation rate and acceptable toxicity [44].

One of the key goals of neoadjuvant therapy is to reduce tumor size and improve the likelihood of complete (R0) resection. Several studies have demonstrated significant tumor shrinkage following neoadjuvant immunotherapy. For example, a study by Uppaluri et al. [16] reported that a group of patients with locally advanced, resectable HNSCC who received neoadjuvant pembrolizumab (an anti-PD-1 antibody) showed significant tumor regression, facilitating easier surgical resection. In many cases, the tumor downstaging allowed for less extensive surgery, reducing the need for mutilating procedures such as mandibulectomy or glossectomy.

Patients with histopathologically confirmed locally advanced squamous cell carcinomas and cases of P16-positive oropharyngeal carcinoma at clinical stage II–III, or non-P16-positive oropharyngeal carcinoma and other HNSCCs at stage III–IV suitable for complete surgical resection were enrolled in a prospective, single-arm clinical trial conducted at a single center by Kai Wang et al. in 2021 [30]. These patients were administered preoperative neoadjuvant therapy, which included 200 mg of pembrolizumab along with 75 mg/m^2^ of cisplatin and 175 mg/m^2^ of paclitaxel. Following this treatment, they underwent surgery and received postoperative adjuvant therapy. Surgery was performed on the patients approximately 4 weeks after their last neoadjuvant treatment, with an average interval of 34.9 days. The main postoperative complication was delayed wound healing, occurring in 22.7% (5 out of 22) of patients, but there were no severe perioperative complications or deaths. All complications were successfully managed with conservative treatments. A total of eight patients (36.4%) achieved a pathological complete response (pCR). Among the remaining 14 patients who did not achieve pCR, five had residuals in both the primary lesions and cervical lymph nodes, one had a pCR only in the primary lesion, and eight had a pCR only in the lymph nodes. The overall pCR rate for cervical lymph nodes was 71.4% (15 out of 21 patients, with one being clinically node-negative). The pCR rate for primary lesions was 40.6% (9 out of 22 patients). Of the 13 patients with residual primary lesions, one had only carcinoma in situ, while six showed scattered lesions with shallow depth of invasion and severe atypical hyperplasia. The rate of major pathological response (MPR) was 54.5% (12 out of 22 patients), with 17 patients having a lower pathological stage after surgery. Only one patient had a pathology-confirmed prevertebral fascia invasion, leading to a T4b vs. T2 classification before surgery. In the nine patients with hypopharyngeal carcinomas and unilateral vocal cord fixation, the MPR rate was 55.6% (5 out of 9 patients). Only two patients with hypopharyngeal carcinoma required total laryngectomy. Post-operative pathological high-risk factors included vascular tumor thrombi (in two patients) and extranodal extension (in two patients). All surgeries resulted in R0 resections, with no positive margins found in postoperative pathology exams. Three patients had residual tumor at the initial intraoperative margin, but after extensive resection, no tumor remained at the margins.

To identify factors linked to pCR, correlation analyses were conducted between pCR and patients’ demographic and clinical characteristics, as well as their disease stage prior to neoadjuvant therapy. CPS and P16 status were used as markers for these statistical analyses. Of the patients, 15 (68.2%) had CPS > 5 in their primary lesion biopsies, and this was significantly correlated with pCR (*p* = 0.015). Additionally, pCR was correlated with pre-treatment tumor stage (*p* = 0.028). However, no correlations were found between pCR and the diameter of the primary lesion, cervical lymph nodes, or P16 status [45].

## 3. Conclusions

Although neoadjuvant immunotherapy holds immense promise, challenges such as identification of predictive biomarkers, management of immune-related adverse events and optimization of treatment regimens remain significant hurdles. Harnessing the body’s immune system to target malignant cells can be an innovative strategy that improves clinical outcomes, redefining standards of care in head and neck cancer. Moreover, further investigations are needed to elucidate the optimal sequencing and combination strategies involving immunotherapy and other treatment modalities.

Neoadjuvant immunotherapy, other than providing an increased ORR (close to 40% in some studies), is expected to allow for less radical and equally effective surgery. This latter feature can significantly help in locally advanced settings. In addition, association of neoadjuvant immunotherapy with radiotherapy can also significantly enhance the so-called abscopal effect induced by some types of radiotherapy (altered fractionations or hadron-therapy). Non-conventional (altered) radiotherapy fractionations, as well as hadron-therapy (proton therapy and carbon-ion therapy), are able to induce necroptosis more than apoptosis. Necroptosis is a cell-death process that presents intermediate characteristics between apoptosis and necrosis, but above all, it is accompanied by inflammation, massive release of tumor-associated antigens (TAAs) and damage-associated molecular pathways (DAMPs). DAMPs and TAAs immediately recall the dendritic cells (DCs) on site, triggering the adaptive, cell-mediated immune response against cancer cells [46].

As ongoing research endeavors to unravel the complexities of tumor immunology and therapeutic resistance, neoadjuvant immunotherapy stands at the forefront of a transformative era in cancer treatment, offering renewed optimism for patients facing this challenging disease.

## Data Availability

Informed consent was obtained from all subjects involved in this study.
